# Towards the Clinical Translation of 3D PLGA/β-TCP/Mg Composite Scaffold for Cranial Bone Regeneration

**DOI:** 10.3390/ma17020352

**Published:** 2024-01-10

**Authors:** Yongsen Zhou, Jingqi Hu, Binhan Li, Jingjing Xia, Ting Zhang, Zhuo Xiong

**Affiliations:** 1 Biomanufacturing Center, Department of Mechanical Engineering, Tsinghua University, Beijing 100084, China; yongszhou@mail.tsinghua.edu.cn (Y.Z.); lbh22@mails.tsinghua.edu.cn (B.L.); xiajj19@mails.tsinghua.edu.cn (J.X.); t-zhang@tsinghua.edu.cn (T.Z.); 2 Biomanufacturing and Rapid Forming Technology Key Laboratory of Beijing, Beijing 100084, China; 3 Biomanufacturing and Engineering Living Systems, Innovation International Talents Base (111 Base), Beijing 100084, China; 4 National Engineering Research Center of Neuromodulation, School of Aerospace Engineering, Tsinghua University, Beijing 100084, China; hujq22@mails.tsinghua.edu.cn

**Keywords:** composite scaffolds, cranial bone regeneration, low-temperature deposition manufacturing, PLGA

## Abstract

Recent years have witnessed the rapid development of 3D porous scaffolds with excellent biocompatibility, tunable porosity, and pore interconnectivity, sufficient mechanical strength, controlled biodegradability, and favorable osteogenesis for improved results in cranioplasty. However, clinical translation of these scaffolds has lagged far behind, mainly because of the absence of a series of biological evaluations. Herein, we designed and fabricated a composite 3D porous scaffold composed of poly (lactic-co-glycolic) acid (PLGA), β-tricalcium phosphate (β-TCP), and Mg using the low-temperature deposition manufacturing (LDM) technique. The LDM-engineered scaffolds possessed highly porous and interconnected microstructures with a porosity of 63%. Meanwhile, the scaffolds exhibited mechanical properties close to that of cancellous bone, as confirmed by the compression tests. It was also found that the original composition of scaffolds could be maintained throughout the fabrication process. Particularly, two important biologic evaluations designed for non-active medical devices, i.e., local effects after implantation and subchronic systemic toxicity tests, were conducted to evaluate the local and systemic toxicity of the scaffolds. Additionally, the scaffolds exhibited significant higher mRNA levels of osteogenic genes compared to control scaffolds, as confirmed by an in vitro osteogenic differentiation test of MC3T3-E1 cells. Finally, we demonstrated the improved cranial bone regeneration performance of the scaffolds in a rabbit model. We envision that our investigation could pave the way for translating the LDM-engineered composite scaffolds into clinical products for cranial bone regeneration.

## 1. Introduction

Cranial bone defects can be caused by congenital deformities or acquired injuries like trauma and cancer, posing a huge burden on patients economically and psychosocially [1]. Effective reconstruction of these defects still represents a common clinical challenge despite the great advancement achieved in cranioplasty. The outcome of cranial restoration is highly dependent on the specific treatment, as well as other factors including surgical skills, tissue environment surrounding the defect, and characteristics of the defect. Traditionally, natural bones harvested from a patient’s skeleton (autograft), donor (allograft), and different species (xenograft) were grafted to the defect area to restore cranial functionality and aesthetic appearance when defects are larger than critical sizes [2]. Autograft has long been regarded the gold standard due to its histocompatibility, nonimmunogenicity, osteoconductivity, and osseointegration, yet its application has hugely been hampered by many shortcomings such as donor site morbidity, severe bone resorption, limited graft availability, and processing difficulty [3]. 

The emergence of bone tissue engineering (BTE) has offered an alternative strategy for cranial bone regeneration, where the synergistic combination of cells, bioactive molecules, and biomaterials induces the formation of new bone tissues by eliminating the risks associated with traditional bone grafts [4,5]. BTE is characterized by functional scaffolds that mimic the extracellular matrix (ECM), assisting the proliferation, differentiation, and biosynthesis of cells residing atop. To achieve favorable cranial bone reconstruction, scaffolds are required to meet several primary design criteria, encompassing (1) good biocompatibility to reduce immune responses, (2) sufficient mechanical strength for brain protection, (3) proper porosity and pore sizes for nutrients and waste transfer, tissue ingrowth, and vascularization, and (4) controlled degradability so that the resorption rate of scaffolds matches the rate of bone formation [6,7,8,9,10,11]. The past few decades have witnessed the rapid development of scaffolds for BTE, owing to the increasing abundance of biomaterials and continuous emergence of new fabrication techniques [10,12]. Different categories of biomaterials have been explored to construct BTE scaffolds for cranial bone regeneration including metals, ceramics, polymers, hydrogels, and composites, among which bioactive composites have demonstrated enhanced bone repairing capabilities as they combine the advantages of individual groups of materials, providing greater flexibility in tuning the biological, mechanical, and structural properties of the engineered scaffolds [13,14,15,16,17,18,19,20,21,22,23,24,25]. For example, Kim et al. have developed a multifunctional hybrid scaffold by immobilizing a bioactive nanocomplex (NC) composed of bone morphogenetic protein 2 (BMP2) and polydeoxyribonucleotide (PDRN) on a preformed scaffold (PME) comprising poly(lactic-co-glycolic) acid (PLGA), ricinoleic acid modified magnesium hydroxide (mMH), and bone extracellular matrix (bECM) [17]. The engineered PME/NC scaffold was examined in a critical-size rat calvarial model, exhibiting outstanding performance in terms of anti-inflammation, angiogenesis, and osteogenesis. In another study, Wang et al. developed a biomimetic composite scaffold by coating a self-assembled glycopeptide hydrogel (GR^gel^) obtained by Schiff’s base reaction between glucomannan and a RADA16 peptide on a 3D-printed scaffold composed of polycaprolactone and nano hydroxyapatite (PCL/nHA) [26]. The ECM-mimicking GR^gel^ not only promotes the proliferation and osteogenic differentiation of endogenous bone mesenchymal stem cells (BMSCs), but also induces macrophage M2 polarization and M2-BMSCs crosstalk, endowing the PCL/nHA@ GR^gel^ scaffold with osteo-immunomodulation and bone regeneration properties, as verified in a critical-size rat skull model. To engineer biomaterials into scaffolds, a large variety of fabrication techniques have been explored, ranging from traditional modalities such as gas foaming, particulate leaching, freeze drying, phase separation, and electrospinning to new modalities exemplified by 3D printing [12,19,27,28,29]. As a newly invented green rapid prototyping method, low-temperature deposition manufacturing (LDM) has demonstrated promising results in scaffold fabrication for BTE [14,30,31,32]. In a typical process of LDM, polymers are dissolved into organic solvents at room temperature and blended (if necessary) with other functional substances such as inorganic fillers and then extruded from a moving nozzle into a low-temperature refrigerator to fabricate frozen scaffolds with computer-designed interconnected macropores. Because of the temperature drop, liquid–liquid and solid–liquid phase separation occurs in the frozen scaffolds. The as-fabricated scaffolds are then subjected to lyophilization, by which the embedded solvents are removed, leaving interconnected micropores [33]. Compared to the commonly used extrusion-based printing methods such as fused deposition modeling (FDM), LMD does not require the melting of materials at high temperatures, which helps to maintain the bioactivity of biomaterials. Moreover, the unique feature of phase separation during printing allows for the precise controllability of the pore size, geometry, and pore interconnectivity of LDM [27]. In a typical study, Yu et al. have employed LDM to fabricate highly porous (90%) PLGA/TCP (tricalcium phosphate) composite scaffolds with interconnected macro- (360 μm) and micro- (3–5 μm) pores and investigated the potential of repairing cranial defects in a goat model [14]. More recently, Shi et al. have fabricated bioactive PLGA/TCP scaffolds incorporating phytomolecule icaritin (ICT, a therapeutic small molecule) by LDM [34]. The resulting PLGA/TCP/ICT scaffolds exhibited macropores of 450 μm and micropores of 20 μm, respectively, and a porosity of 70%, greatly facilitating the bone ingrowth. Despite the encouraging results of composite scaffolds fabricated by LDM [14,34,35], patients with cranial bone defects still do not have access to these LDM-engineered scaffolds in the clinical setting. Such unmet needs call for more preclinical investigations, particularly a series of biological evaluations designed for non-active medical devices. 

In this study, we investigated the potential of a 3D composite scaffold fabricated by LDM for cranial bone regeneration. To achieve favorable regeneration results, PLGA, β-TCP, and Mg were chosen as the major components of the scaffold. The morphology, porosity, and mechanical properties of the LDM-engineered PTM scaffolds were characterized by scanning electron microscope (SEM), mercury intrusion porosimetry, and uniaxial compression, respectively. The composition of the PTM scaffolds was analyzed by inductively coupled plasma optical emission spectrometry (ICP-OES), Fourier transform infrared (FTIR), and X-ray powder diffraction (XRD). In particular, subchronic systemic toxicity and local effects after implantation animal tests (two important biologic evaluation of non-active medical devices) were conducted to evaluate the biological safety of the scaffolds. In vitro osteogenic differentiation tests of different scaffolds were also performed to verify the effectiveness of promoting osteogenesis of the PTM scaffolds. Finally, the in vivo capability of promoting cranial osteogenesis of the PTM scaffolds was validated in a rabbit model. The overall performance of the PTM scaffolds has ascertained the advantages and benefits of LDM-engineered composite scaffolds for the effective repairing of cranial bone.

## 2. Materials and Methods 

### 2.1. Materials

Medical-grade PLGA (70:30, 1.0 ± 0.2 dL/g, Sichuan Dikang Sci-Tech Pharmaceutical Co., Ltd., Chengdu, China), β-TCP (grain size 3 μm, Beijing Deke Daojin Science and Technology Co., Ltd., Beijing, China), Mg powders (100–200 mesh size, Sinopharm Chemical Reagent Co., Ltd., Shanghai, China), and 1, 4-dioxane (99%, ACS reagent, J&K Scientific, Beijing, China) were used as received. 

### 2.2. Scaffold Fabrication

The scaffold fabrication was based on previous studies with minor modifications [30,36]. First, PLGA was dissolved in 1, 4-dioxane at a concentration of 0.15 g/mL using a magnetic stirrer. Then, β-TCP and Mg powders were added to the PLGA solution at a mass ratio of 16:1:3 (PLGA:β-TCP:Mg), followed by vigorous stirring. The as-prepared paste was extruded layer by layer at a thickness of 100 μm and distance of 1.1 mm by a low-temperature deposition 3D-printing machine (ALPHA-BP 11, SunP Biotech, Beijing, China) with a diameter 400 μm spinneret to form a cubic porous scaffold measuring 10 mm × 10 mm × 10 mm under low temperature of −25 °C. Finally, the scaffolds were lyophilized in a freeze dryer (LGJ-10FD, Beijing Songyuan Huaxing Technology Develop Co., Ltd., Beijing, China) for at least 14 days under a vacuum pressure of 50 Pa to remove the solvent 1,4-dioxane, followed by EO sterilization. For the small-sized scaffolds used in the animal studies, larger scaffolds were printed first and then cut into small samples manually.

### 2.3. In Vitro Evaluation of Scaffolds

#### 2.3.1. Morphology

The morphology of printed scaffolds was observed by field emission scanning electron microscope (SEM, Zeiss Supra55, Carl Zeiss, Jena, Germany). Briefly, the scaffold was first lyophilized and then fractured in liquid nitrogen, followed by sputtering a thin layer of gold to provide electrical conductivity. The average pore size of the scaffold was determined from the SEM images. 

#### 2.3.2. Porosity

The Auto Pore V 9600 mercury intrusion porosimetry was used to test the pore structure of samples according to ISO 15901-1:2005 [37], IDT. Before testing, samples were dried in the oven at 50 °C for 24 h. The samples were intruded in a low-pressure porosimeter with pressure up to 0.51 psia (corresponding to 125 um) first and then moved in a high-pressure porosimeter with pressure up to 60,000 psia (corresponding to 3 nm). The equilibration time of the low-and high-pressure stage is 20 s and 30 s, respectively. The pore diameter (size) *d* can be calculated by the Washburn equation, $d=-\frac{4\gamma \cos\theta }{P}$, where *P* is the applied pressure, γ is the surface tension of mercury (0.48 N/m), and θ is the contact angle between mercury and the pore surface (130°). Note that the values of γ and θ were taken according to the relevant suggestion in ISO 15901-1:2005, IDT.

#### 2.3.3. Composition

The elemental composition of the scaffold was determined by inductively coupled plasma optical emission spectrometry (ICP-OES, AVIO 200, Perkinelmer, Waltham, MA, United States of America). Briefly, samples were weighed and dissolved in a mixture of nitric acid (6 mL), hydrochloric acid (3 mL), and hydrogen peroxide (1 mL) at 150 °C. After complete dissolving, the solution was diluted and analyzed by ICP-OES. The composition of the scaffold was verified by Fourier transform infrared with the attenuated total reflectance accessory (FTIR-ATR, Nicolet iS20, Thermo Fisher Scientific, Waltham, MA, United States of America) at a resolution of 4 cm^–1^, 64 scans, in the range of 4000–500 cm^–1^. The existence of β-TCP in the scaffold was analyzed by X-ray powder diffraction (XRD, D8 ADVANCE, Bruker, Germany).

#### 2.3.4. Mechanical Property

The mechanical properties of scaffolds were characterized by the uniaxial compression test according to ISO 844:2014 [38]. Briefly, the scaffolds were cut into cubes measuring 10 × 10 × 10 mm^3^ and compressed by the universal testing machine (CTM210, Xie Qiang Instrument Manufacturing Co., Ltd., Shanghai, China) with a load of 1000 N and speed of 1 mm/min. Three samples in each group were tested and the load–displacement curves were obtained to calculate the compressive modulus and compressive strength of the scaffolds.

#### 2.3.5. Osteogenic Differentiation

The osteogenic differentiation of scaffolds was assessed by culturing MC3T3-E1 cells (Subclone 14, CRL-2594, American Type Culture Collection, Manassas, VA, USA) with the leaching solution of the scaffolds and quantifying the mRNA transcript levels of osteogenic specific genes according to the literature with minor modifications [39]. Briefly, the leaching solution of the scaffolds was prepared by incubating the scaffolds in the MEM Alpha medium (α-MEM, Gibco, Waltham, MA, USA, A10490-01) containing 10% FBS (Gibco, Waltham, MA, USA, 10099-141) and 1% PS (Hyclone, Marlborough, MA, USA, SV30010) at 37 °C for 72 h. Then, the leaching solution was diluted 10 times with the α-MEM solution supplemented with 10 mmol/L β-Glycerophosphate disodium salt (Sigma-Aldrich, St. Louis, MO, USA, G9422), 50 μg/mL ascorbic acid (Sigma-Aldrich, St. Louis, MO, USA, A4544), and 10 nmol/L dexamethasone (Sigma-Aldrich, St. Louis, MO, USA, D4902) to reduce the cytotoxicity of Mg [40]. For the in vitro osteogenic differentiation, MC3T3-E1 cells at a density of 4 × 10^4^ were seeded in 12-well plates and incubated with α-MEM basal medium for 72 h. Then, the basal medium was changed to the diluted leaching solution and we continued incubating the cells for 7 days. The culturing medium was changed every two days. Then, the cells were collected, and the total RNA was extracted using the TRIZOL reagent (H10318, TransGen Biotech, Beijing, China), followed by reverse transcribing the total mRNA into cDNA using the FastKing-RTSuperMix (KR118-02, TIANGEN BIOTECH (Beijing) Co., Ltd., Beijing, China). cDNA template and specific primers (Table S17) were synthesized by Shanghai Generay Biotechnology Co., Ltd., Shanghai, China. The mRNA levels of all genes were normalized to the target gene (GAPDH). 

### 2.4. In Vivo Evaluation of Scaffolds

#### 2.4.1. Subchronic Systemic Toxicity

According to ISO 10993-11:2017 [41], 40 SD rats (male and female, weight range: 171.9~236.6 g) were selected and randomly divided into two groups: experimental group (10 of each sex) and control group (10 of each sex). Briefly, anesthesia was performed by intraperitoneal injection of 1% pentobarbital sodium solution before surgery. After anesthesia, the outer femoral coat was removed, the skin of the surgical area was routinely disinfected, and a surgical towel was spread. An incision was made in the femur of the animal along the long axis of the femur, and the subcutaneous tissue, muscle, and periosteum were separated layer by layer to expose the cortical bone. Under the conditions of a low-speed water spray, a hole was drilled into the bone with a Φ2 mm drill, and the scaffold was implanted into the hole, gently pressurized so that the scaffold was flat or slightly protruded from the hole, and the remaining sample was placed in the periphery of the bone, and the total implantation dose in the femur of both sides was 10 samples (about 0.059 g). After implantation, the layers of tissue were sutured, the skin was closed, and the wound and surgical area were treated with iodophor. Control animals were given the same surgical treatment, but no scaffolds were placed. This experiment cycle totaled 90 days and each animal was weighed every 7 days. Clinical observation was performed by recording the general state and toxicity manifestations of the animals in each group, including the time of occurrence and severity and duration of changes in the skin, coat, eyes, and mucous membranes, as well as changes in respiration, circulation, autonomic and central nervous systems, somatic movements, and behavioral patterns. Meanwhile, all animals were weighed and fasted for 12~18 h prior to necropsy. On the day of necropsy, after anesthetizing the animals, blood was collected from the abdominal aorta for complete blood cell count and clinical chemistry tests. Regarding the necropsy, the wet weight of major organs was assessed and pathologic changes in each organ were recorded, including heart, liver, spleen, lungs, kidneys, adrenal glands, gonads, brain, thymus, stomach, etc.

#### 2.4.2. Local Effects after Implantation

According to ISO 10993-6:2016 [42], sixteen Japanese white rabbits (male and female, weight range: 2.43~2.65 kg) were selected. After the animal was anesthetized, the outer coat of the thigh was removed, and the animal was fixed on the operating table with the skin of the surgical area routinely disinfected. Then, a surgical towel was placed, leaving an operating area about 5 cm × 5 cm. In the femur of the animal, an orifice about 3~4 cm long was made along the long axis of the femur. Blunt separation was made along the two muscle bundles, exposing the femur. Periosteum was pushed away with bone peeling, followed by drilling three Φ2 mm holes at the distal, middle, and proximal ends of the femur, respectively. Under the conditions of low-speed water spray, the PTM scaffolds and control scaffolds (purchased from Dikang Biomedical Co., Ltd., Chengdu, China) were implanted in the left and right femur, respectively. The incision was closed, and the muscle, subcutaneous tissue, and skin were sutured in layers. Finally, the wound was disinfected with iodophor. At 1, 4, 13, and 26 weeks, four animals were executed by excess CO_2_, and the femur was isolated and removed. After fixation, sectioning, and decalcification, the PTM scaffolds were not taken out and the control scaffolds were taken out. The tissue blocks were dehydrated with graded alcohol, paraffin-embedded, sectioned, and hematoxylin & eosin (H&E) stained for histopathological observation and evaluation of the material.

#### 2.4.3. Rabbit Model of Cranial Bone Regeneration

Twelve male New Zealand white rabbits (3–4 months old) were purchased from Shandong Ilaike Biotechnology Co., Ltd. (Weifang, China). and quarantined for 6 days prior to experiment. A parietal bone defect in a rabbit model was built to evaluate the in vivo bone regeneration of the composite scaffold. Briefly, the skin on the top of the head of the New Zealand rabbit was peeled 24 h before the implantation. Then, 0.08 mL/kg Shutai ^®^50 and 0.08 mL/kg tachysleep were injected intravenously and intramuscularly injected, respectively, for general anesthesia. After anesthesia, the rabbits were lay prone on the operating table and the operating area was disinfected with iodophor. For the implantation, a middle incision of 5 cm in length on the scalp was first made with surgical instruments, followed by the creation of four bone defects symmetrically distributed on the two sides of the skull suture with a low-speed bone drill. During this procedure, the defects were washed thoroughly with saline and a suction device. Next, two composite scaffolds were press-fitted into the left defect sites (experiment group), whereas no scaffold was implanted in the right defect sites (control group). The implantation was finalized by suturing the scalp, disinfecting the wound with iodophor, and covering with a sterile yarn cloth. After the implantation, the rabbits were injected intramuscularly with penicillin sodium for 3 consecutive days for anti-inflammatory purposes and we carefully watched the healing of the surgical incision to avoid infection. The 12 rabbits were divided into six groups, corresponding to the six post surgery time points: 2 weeks, 4 weeks, 6 weeks, 8 weeks, 10 weeks, and 12 weeks. The rabbits were checked daily, and their weight was recorded weekly. Any suspected observation of local reaction and abnormal behavior during the experimental period was noted. At each time point, two rabbits were sacrificed by an air embolization method and dissected, exposing the implantation site for the assessment of surrounding tissue. Femoral condyle was isolated for micro-CT scanning, histological analysis, and mechanical test.

### 2.5. Statistical Analysis

All the data in this study were expressed as mean ± standard deviation. Statistical analysis was performed with MATLAB software (https://www.mathworks.com/products/matlab.html, 7 January 2024). One-way analysis of variance (ANOVA) followed by Tukey’s multiple comparison test was conducted for the comparison between multiple samples with thresholds of * *p* ≤ 0.05, ** *p* ≤ 0.01, *** *p* ≤ 0.001, and **** *p* ≤ 0.0001. Student’s t-test was conducted for the comparison between two samples with thresholds of * *p* ≤ 0.05, ** *p* ≤ 0.01, *** *p* ≤ 0.001, and **** *p* ≤ 0.0001.

## 3. Results and Discussion 

### 3.1. Design and Fabrication of PTM Scaffolds

**Figure 1:**
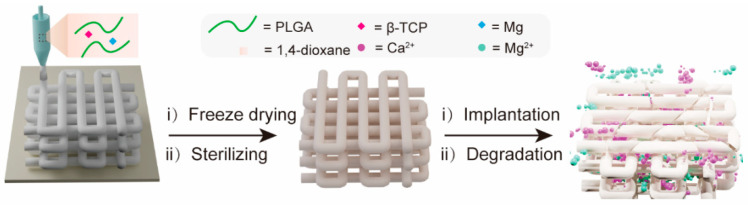
Schematic illustration of the fabrication of PTM composite scaffold by LDM and controlled release of Ca^2+^ and Mg^2+^ after implantation.

Figure 1 schematically illustrates the fabrication of a 3D porous composite scaffold by LDM. This PTM scaffold incorporates three major components, i.e., PLGA, TCP, and magnesium (Mg). PLGA was chosen as the backbone material of the scaffold due to its excellent biocompatibility, adjustable degradability, sufficient mechanical properties, and capability to promote bone regeneration [43]. β-TCP was incorporated into the scaffold to neutralize the acidic degraded products from PLGA while providing calcium and phosphate ion depots to promote new bone formation through osteoinduction [44]. The reasons to incorporate Mg into the scaffold are multifold. Firstly, adding Mg powder as an inorganic filler can enhance the mechanical properties of the scaffold. Secondly, the released Mg ions can also help to neutralize the acidic degraded products from PLGA. Thirdly, Mg ions can not only promote the osteogenic differentiation of MSCs by elevating autophagic activities, but also show angiogenic and anti-inflammatory properties [45,46]. After the PTM scaffold was engineered, it was freeze-dried to remove the cytotoxic 1,4-dioxane, followed by EO sterilizing to eliminate any possible microorganism. When implanted in vivo, the PTM is subject to degradation over time, releasing functional Ca^2+^ and Mg^2+^ that enhance osteogenesis. 

### 3.2. Basic Characterization of PTM Scaffolds

**Figure 2:**
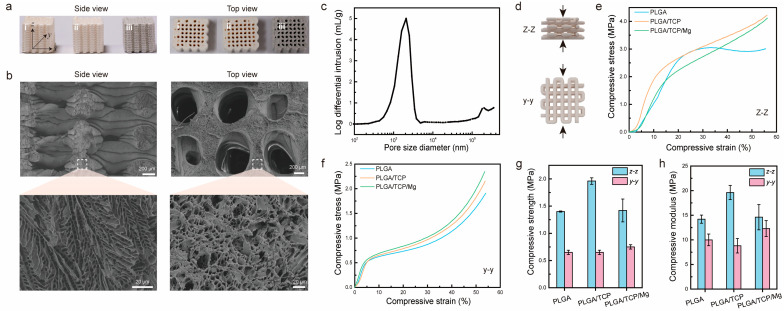
Morphological and mechanical characterization of PTM scaffolds. (**a**) Side view and top view of different scaffolds, i–iii corresponds to PLGA, PLGA/β-TCP, PLGA/β-TCP/Mg scaffolds, respectively. (**b**) SEM images of PTM scaffolds. (**c**) Porosity measurement of PTM scaffolds. (**d**) Illustration of the compression test from two directions. (**e**,**f**) Stress and strain curves of different scaffolds under z–z compression and y–y compression. (**g**) Compressive strength of different scaffolds. (**h**) Compressive modulus of different scaffolds.

First, we characterized the macrostructure of PTM scaffolds together with PLGA scaffolds and PLGA/β-TCP (PT) scaffolds. Figure 2a shows photographs of the three different scaffolds after sterilization. Clearly, it was observed that the scaffolds were stacked layer by layer from the side view, generating micrometer-sized gaps or pores. Almost all the larger open pores (centimeter-size) could be observed from the top view, except some closed pores. It could also be observed that the color of the PTM scaffolds is darker than the other two scaffolds, owing to the incorporation of Mg. Next, we characterized the microstructure of PTM scaffolds by SEM. As shown in Figure 2b, the surface of the extruded filament is rough and interconnected micro-pores with sizes ranging from 2 μm to 10 μm could be observed inside the filament (side view), resembling the microstructure or cancellous bone. Meanwhile, macro-pores with sizes ranging from 700 μm to 1000 μm could be observed (top view), which is well correlated with the printing parameter (1100 μm). To quantify the porosity, mercury intrusion porosimetry was conducted. As shown in Figure 2c, the pore size mostly dwells in the range of 1–4 μm, which facilitates neovascularization. The calculated porosity of the PTM scaffolds is 63 ± 1%, comparable to that of cancellous bone (50–90%). The combination of micro- and macro-pores with proper pore size, appropriate porosity, and excellent pore interconnectivity have validated the advantages of LDM for the fabrication of 3D porous scaffolds.

To ensure effective bone repairing, the scaffolds should exhibit sufficient mechanical strength. Since the scaffolds are anisotropic, mechanical properties might vary from different directions. Figure 2d illustrates the compression test of scaffolds at the y–y and z–z directions. As shown in Figure 2e, when compressed from the z–z direction, the PLGA scaffold exhibited a yield stress of 3 MPa at a compressive strain of 25%, whereas the PT and PTM scaffolds presented increased compressive stress as the strain increased. When compressed from the y–y direction (Figure 2f), all the scaffolds demonstrated a similar trend, i.e., linear increase in stress at strain below 10% and exponential increase in stress at strain above 40%. To further compare the mechanical properties of different scaffolds, we calculated the compressive strength (defined as the maximum stress at a strain below 10%) and compressive modulus (defined as the stress over strain in the linear range). As shown in Figure 2g, the difference in compressive strength of different scaffolds at different directions varied. Specifically, for the z–z direction, the PT scaffold presented the highest compressive strength (1.96 MPa), whereas the PLGA and PTM scaffolds exhibited comparable compressive strength (1.4 MPa). However, for the y–y direction, the PLGA and PT scaffolds exhibited comparable compressive strength (0.65 MPa), which is lower than that of the PTM scaffold (0.75 MPa). In the case of the compressive modulus, the results were more complicated (Figure 2h). Although the PT scaffold presented the highest compressive modulus (19.6 MPa) at the z–z direction, its compressive modulus was the lowest (8.8 MPa) at the y–y direction. Theoretically, adding inorganic fillers (e.g., β-TCP and Mg) into the PLGA scaffold would result in improved mechanical properties. However, the PTM scaffold in our study did not demonstrate enhanced mechanical properties as reported in other studies [36,47]. Such discrepancy is likely to be attributed to the printing parameters and morphological differences of the Mg powder used in different studies. Specifically, the Mg powder used in our study exhibited irregular shapes (Figure S1a), whereas other studies used spherical Mg powder (Figure S1b). In spite of this unexpected result, the mechanical properties of the anisotropic PTM scaffolds met the basic requirements of scaffolds for BTE.

Since the mass ratio of the three major components in the PTM scaffolds determines the mechanical properties and the performance of bone repairing, it is important to maintain the mass ratio during LDM and subsequent freeze-drying and sterilization. To verify this, we detected the concentration of Ca^2+^ and Mg^2+^ by ICP-OES and deduced the real mass ratio in the final PTM scaffolds. As shown in Figure S2a, 1.97% Ca^2+^ and 14.49% Mg^2+^ were detected in the PTM scaffolds, corresponding to 5.08% β-TCP and 14.49% Mg. Obviously, the original mass ratio in the paste (i.e., 5% β-TCP, 15% Mg) was well maintained, highlighting the advantages of LDM in obtaining scaffolds with predetermined composition. The PTM scaffolds were further analyzed by FTIR to verify the existence of the three major components. As depicted in Figure S2b, the characteristic peaks at 2996 cm^−1^ (stretching vibration of -CH_3_), 2946 cm^−1^ (stretching vibration of C-H), 1748 cm^−1^ (stretching vibration of C=O), 1455 cm^−1^ (bending vibration of C-H), 1384 cm^−1^ (bending vibration of C-H), 1181 cm^−1^ (stretching vibration of C-O), and 1084 cm^−1^ (stretching vibration of C-O) are from PLGA, while the characteristic peaks at 1129 cm^−1^ (stretching vibration of P-O), 1044 cm^−1^ (stretching vibration of P-O), 964 cm^−1^ (bending vibration of P-O), and 606 cm^−1^ (bending vibration of O-P-O) are from β-TCP. Next, the crystal phase of β-TCP and Mg in the PTM scaffolds was confirmed by XRD. As shown in Figure S2c, the diffraction peaks of both β-TCP and Mg were sharp and intense, indicating their highly crystalline nature. No impurity peaks were observed, confirming the high purity of the two components. 

To translate the PTM scaffolds into clinical products, it is vital to ensure the scaffolds are not cytotoxic. Therefore, the cytotoxic solvent of 1,4-dioxane used for paste preparation and pore construction during lyophilization should be reduced to a safe level (i.e., below 400 ppm). We found that the lyophilizing time and sample temperature greatly affected the concentration of residual 1,4-dioxane. Specifically, after three days of lyophilization, the residual 1,4-dioxane could be as high as 8000 ppm, which was reduced to 650 ppm at Day 10 and further reduced to 165 ppm at Day 14 (Figure S3a). To speed up the lyophilization process, we increased the sample temperature and found that an increase of only 1 °C could lead to a significant drop in residual 1,4-dioxane (from 438 ppm to 277 ppm, Figure S3b). 

### 3.3. Biological Evaluation of PTM Scaffolds

**Figure 3:**
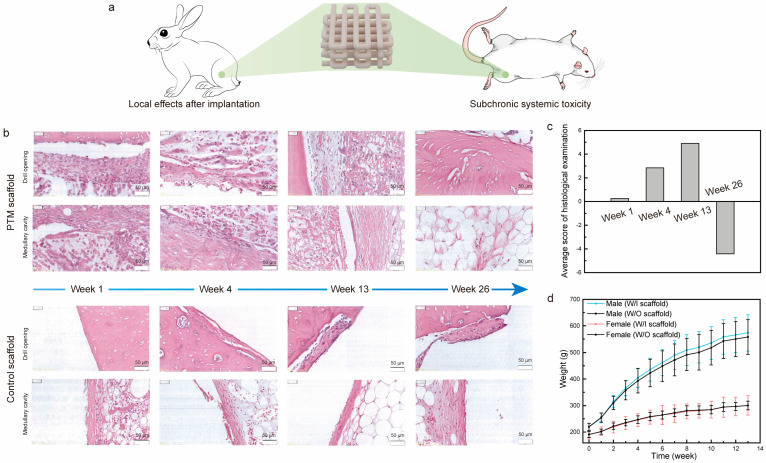
(**a**) Illustration of the implantation of scaffolds in the local and systemic toxicity evaluation tests. (**b**) H&E staining images of PTM and control scaffolds implanted in the femur of rabbits at different time intervals. (**c**) Histological scoring of the PTM scaffolds at different time intervals. (**d**) The weight change of SD rats in the subchronic systemic toxicity tests.

To examine the local and systemic toxicity of the PTM scaffolds, we analyzed the local effects after implantation and subchronic systemic toxicity tests, which are two important tests for the biological evaluation of class III medical devices (Figure 3a and Figure S4). As for the local toxicity evaluation of scaffolds, commercially available scaffolds were taken as control samples. The gross observation of the exposed femur after separating the tissue did not find any femoral breaks, abscesses, hemorrhages, or other abnormalities. Figure 3b shows the H&E staining images of scaffolds at different time intervals of implantation. Specifically, one week after implantation: (1) it was observed in both groups that the periphery of the implant material was encapsulated by a continuous cellular layer (thickness in the range of 20–660 μm in the PTM groups and 6–680 μm in the control group) mainly composed of fibroblasts, fibrocytes, and fibers, and neoplastic trabeculae were visible in some areas of the implant–medullary cavity interface; (2) a very small number of polymorphonuclear leukocytes and macrophages were seen in the cellular layer in both groups, and giant cells and lymphocytes were seen in the PTM and control groups, respectively; (3) scattered polymorphonuclear leukocytes and very few macrophages were seen in the cellular layer, and very few giant cells and lymphocytes were seen in the PTM and control groups, respectively. Similarly, four weeks after implantation: (1) the periphery of the implant material remained to be encapsulated by a continuous cellular layer (thickness in the range of 10–430 μm in the PTM groups and 5–260 μm in the control group) mainly composed of fibroblasts, fibrocytes, and fibers in both groups; (2) some area of the implant–medullary cavity interface was closed by trabeculae (both groups), and particularly, the drill opening at the implant–bone interface was closed by trabeculae in the PTM group; (3) a very small number of polymorphonuclear leukocytes, lymphocytes, and macrophages were seen in the cellular layer in both groups, and giant cells were seen in the PTM group only; (4) scattered polymorphonuclear leukocytes and a very small number of lymphocytes and macrophages were seen in the cell layer of both groups, and giant cells were seen in the PTM group only. At longer times of implantation (13 weeks), the difference between the two groups became obvious: (1) the drill opening at the implant–bone interface was closed by new bone in both groups; (2) the periphery of the implant material continued to be encapsulated by a continuous cellular layer (thickness in the range of 10–320 μm in the PTM groups and 4–220 μm in the control group) mainly composed of fibroblasts, fibrocytes, and fibers in both groups, and neoplastic trabeculae were seen in some areas of the implant–medullary cavity interface in the PTM group; (3) the implant was infiltrated with a large number of polymorphonuclear leukocytes and some giant cells; (4) a very small number of polymorphonuclear leukocytes and lymphocytes were seen in the cellular layer in both groups, and macrophages and giant cells were seen in the PTM group only; (5) scattered polymorphonuclear leukocytes and a very small number of lymphocytes, macrophages, and giant cells were seen in the cell layers of the PTM group. At the endpoint of implantation (26 weeks), it was observed that: (1) the drill opening at the implant–bone interface was fully closed by new bone in both groups; (2) in 8 out of 12 sections of PTM group, the bone marrow tissue was complete and no obvious implant material was seen, while in 4 out of 12 sections, some residual fibrous tissue was visible (thickness in the range of 15–140 μm), mainly composed of fibroblasts, fibrocytes, and fibers; (3) in 10 out of 12 sections of the control group, residual cellular layer (thickness in the range of 7–120 μm) was visible at the implant–bone interface and implant–medullary cavity interface, mainly composed of fibroblasts, fibrocytes, and fibers.

Based on the grading scales for inflammation and histology (Tables S1 and S2), the average score of histological examination was calculated. As shown in Figure 3c, the semi-quantitative histopathological score was 0.25 at week 1, indicating that the PTM scaffolds did not initiate any irritation after implantation (based on the criteria in Table S3). At week 4 and week 13, the histopathological scores were 2.84 and 4.91, respectively, indicating that the PTM scaffolds initiated only mild irritation. At week 26, the histopathological score was reduced to −4.42 (taken as 0), which means that the mild irritation was fully eliminated with the longer implantation time as the scaffolds degraded. Detailed calculation of the histopathological score can be found in Tables S4–S7. Therefore, testing of local effects after implantation has justified the local non-toxicity of the PTM scaffolds.

Next, we examined the systematic toxicity of the PTM scaffolds by the subchronic systemic toxicity test. All the SD rats involved in the test behaved normally and no symptoms of toxicity caused by implants were observed throughout the experiment. Body weight recording shows that there was no significant difference between the PTM group and the blank group (Figure 3d). By the end of experiment, it was observed that (1) the SD rats in the PTM group exhibited higher WBC (*p* < 0.01), lower NEUT (*p* < 0.05), and higher LYMPH (*p* < 0.05) in the complete blood cell count (Table S8), and (2) the GLU of SD rats in the PTM group was lower (*p* < 0.01) than the blank group (Table S9). However, such differences were acceptable since all the values were within the baseline level and did not indicate any clinical significance. Additionally, other items in the complete blood cell count, clinical chemistry tests, and organ coefficient (Table S10) did not show any significant difference between the PTM and blank groups. Meanwhile, no obvious injury or damage was found in either group during the gross observation. Therefore, it was believed that the PTM scaffolds did not initiate subchronic systemic toxicity.

### 3.4. Osteogenic Differentiation of MC3T3-E1 Cells with Scaffolds

**Figure 4:**
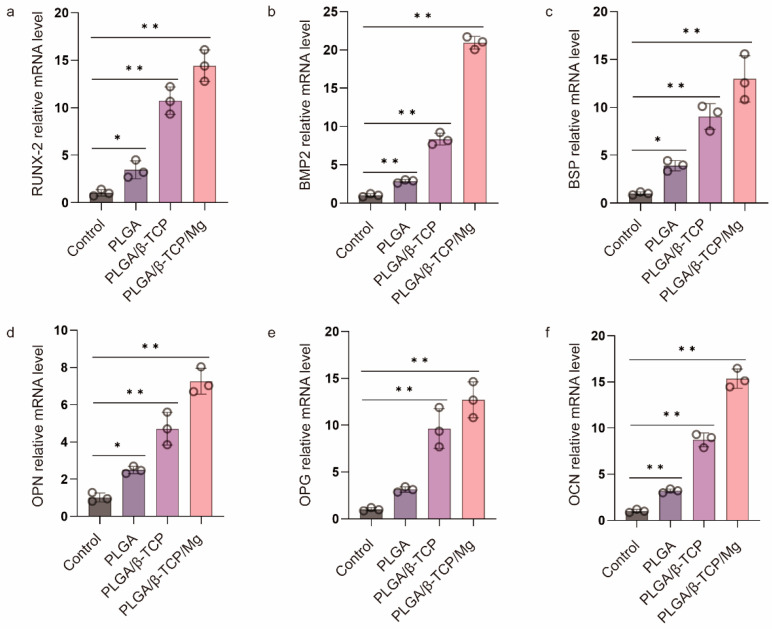
RT-qPCR analysis of (**a**) RUNX-2, (**b**) BMP 2, (**c**) BSP, (**d**) OPN, (**e**) OPG, and (**f**) OCN gene expression of MC3T3-E1 cells after culturing in the osteogenic medium for one week. * *p* ≤ 0.05, ** *p* ≤ 0.01.

Before evaluating the in vivo cranial bone repairing performance of the PTM scaffolds, we verified its in vitro osteogenic differentiation by culturing the osteoblast MC3T3-E1 cells with the α-MEM medium containing the leaching products of scaffolds and quantifying the expression of osteogenic genes via RT-qPCR. PLGA and PLAG/β-TCP scaffolds were also included for comparison. The mRNA expression of six typical osteogenic genes was investigated including Runt-related transcription factor 2 (RUNX-2), bone morphogenetic protein-2 (BMP2), bone sialoprotein (BSP), osteopontin (OPN), osteoprotegerin (OPG), and osteocalcin (OCN). Figure 4 shows that the PLGA scaffolds presented a low level of osteogenic gene expression, whereas the PT and PTM scaffolds exhibited significantly higher levels, confirming the osteogenic potential of β-TCP and Mg. Furthermore, all six osteogenic-related genes were significantly upregulated in the PTM scaffolds compared to the PM scaffolds, indicating the osteogenic potential of Mg [48]. Specifically, RUNX-2 (an essential transcription factor for osteoblast differentiation, matrix production, and mineralization during bone formation [49]) and BMP2 (an early marker of bone formation and a signal molecule to promote alkaline phosphatase expression) were significantly upregulated in the PTM scaffolds (Figure 4a,b), indicating that PTM scaffolds could induce osteogenic differentiation of MC3T3-E1 cells through the BMP signaling pathway. BSP (a late marker of bone formation) was abundant in the bone healing site, and its upregulation (Figure 4c) could positively impact the osteogenic differentiation of endogenous stromal cells. Also as a late marker of bone formation, OPN contains the acidic domain that interacts with the mineral surface of the extracellular matrix, and its upregulation (Figure 4d) could also promote the mineralization of the inorganic phase and realize cell ossification [50]. OPG is secreted by osteoblasts and osteogenic stromal cells and inhibits bone resorption by binding to the receptor activator of NF-κB (RANK) and halts osteoclast activity, and its upregulation (Figure 4e) could prevent excess bone resorption. OCN (a late marker of bone formation) is the most abundant noncollagenous protein in bone tissue, and its increase (Figure 4f) is associated with an increase in bone mineral density. Taken together, the significant upregulation of these osteogenic genes has justified the advantages of PTM scaffolds in promoting osteogenesis in vitro.

### 3.5. Cranial Bone Regeneration of PTM Scaffolds in a Rabbit Model

**Figure 5:**
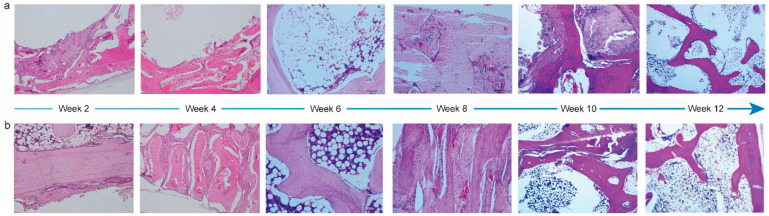
H&E staining images of PTM scaffolds (**a**) and blank scaffolds (**b**) implanted in the cranial bone of rabbits at different time intervals.

To evaluate the performance of PTM scaffolds in repairing and regenerating cranial bone defects, we constructed a rabbit model. It was observed that the average body weight of rabbits prior to and during implantation increased steadily (Figure S5), and all the animals maintained a good mental state, appearance, and normal behaviors. Meanwhile, the gross observation did not find lesions like hematoma, edema, inflammation, and visible cystic cavities in the implantation area. Next, we performed systematic histopathological evaluation by analyzing the H&E staining images. Figure 5 shows the corresponding images of the PTM and blank scaffolds at different time intervals. Two weeks after the implantation, mild levels of inflammatory cell infiltration, fibrous tissue proliferation, osteoblast formation, neoplastic trabecular formation (disordered and unevenly coarsened), and neovascularization were seen at the implantation site in both groups, and multifocal hemorrhage was seen at the implantation site in the blank group. Four weeks after the implantation, mild levels of fibrous tissue proliferation and osteoblast formation were seen in both groups. Mild neovascularization was seen in the blank group, whereas mild inflammatory cell infiltration and neovascularization infiltration were seen in the PTM group. Although mild neoplastic trabecular formation (ordered) was observed in both groups, the neotrabeculae was evenly coarsened in the PTM group, and medium trabecular formation could be observed in the PTM group. Six weeks after the implantation, mild levels of new bone formation, fibrous tissue proliferation, and osteoblast formation were seen in both groups. Medium trabecular formation was observed in only one out of four sections in the blank group, whereas such formation was observed in two out of four sections in the PTM group. Meanwhile, mild adipose tissue proliferation was seen in the PTM group. Eight weeks after the implantation, mild levels of fibrous tissue proliferation, osteoblast formation, and new bone formation (mature osteoblast development, sufficient bone sockets, and well-aligned bone plates) were seen in both groups. Although mild or medium neoplastic trabecular formation could be observed in both groups, the trabeculae was well ordered and evenly coarsened in the PTM group, as compared to the blank group (disordered and unevenly coarsened). Ten weeks after the implantation, mild levels of fibrous tissue proliferation were seen in both groups. Although mild new bone formation was also seen both groups (mature osteoblast development and well-aligned bone plates), there were more bone sockets in the PTM group than the blank group. Additionally, mild osteoblast formation, medium neoplastic trabecular formation (ordered and evenly coarsened), and mild chondrocyte formation were observed in the PTM group. Finally, twelve weeks after the implantation, mild levels of osteoblast formation, neoplastic trabecular formation (ordered and evenly coarsened), and new bone formation (mature osteoblast development, sufficient bone sockets, and well-aligned bone plates) were observed in the blank group, whereas medium neoplastic trabecular formation (ordered and evenly coarsened) and mild new bone formation (mature osteoblast development, sufficient bone sockets, and well-aligned bone plates) were observed in the PTM group. Collectively, the PTM group exhibited higher levels of osteoblast formation, new bone formation, neoplastic trabecular formation, and chondrocyte formation than the blank group. Particularly, the phase of bone scab formation was accelerated in the PTM group, resulting in enhanced repairing of the cranial bone defect. 

The irritation level of the PTM scaffolds after implantation was also semi-quantified by calculating the average score of histological examination (Tables S11–S16). The average histological scores at 2, 4, 6, 8, 10, and 12 weeks post implantation were 3.00, 2.00, 4.75, 3.25, −2.25, and −2.00, respectively. Therefore, the PTM scaffolds only initiated mild irritation at week 2, 6, and 8, and no irritation at week 4, 10, and 12. 

Different from the repairing process of long bones like the femur and tibia, healing the defects of flat bones (e.g., the cranial bone) is more challenging due to the lack of sufficient blood supply and bone marrow [50]. Previously, the bone repairing capability of PTM scaffolds has been examined mostly on long bones and encouraging results have been demonstrated [36]. Whether the PTM scaffolds can promote the healing of flat bone defects or not has not been investigated before. Based on the above results, it is believed that the PTM scaffolds also hold great potential in promoting osteogenesis in flat bones, largely due to the composite composition and overall properties of the scaffolds. 

## 4. Conclusions

In summary, we have engineered a 3D porous composite scaffold using the LDM method. Owing to the unique characteristics of LDM, the engineered scaffold presented micro- and macro-pores with sizes comparable to the cancellous bone. Moreover, the highly interconnected pores could facilitate nutrient and waste exchange, tissue ingrowth, and vascularization. The anisotropic mechanical properties of the scaffolds and the influence of lyophilization time and temperature on the removal of 1,4-dioxane were systematically investigated. More importantly, the local and systemic toxicity of the scaffolds was evaluated by two biological tests necessary for the scaffolds to be translated into clinical products. Attributed to the Ca^2+^ and Mg^2+^ release as the scaffolds degraded, the expression of osteogenic genes was significantly upregulated in the PLGA/β-TCP/Mg scaffolds in comparison to the pure PLAG and PLGA/β-TCP scaffolds. Finally, we demonstrated in a rabbit model that the PLGA/β-TCP/Mg scaffold could improve the outcome of cranial bone regeneration by accelerating the phase of bone scab formation. We believe that this LDM-engineered composite scaffold holds great potential in translating into clinical products for cranial bone regeneration in the near future. 

## Data Availability

Data are contained within the article.

## Supplementary Materials

The following supporting information can be downloaded at: https://www.mdpi.com/article/10.3390/ma17020352/s1, Figure S1: Comparison of morphological appearance of Mg powders used in this study and other studies. Figure S2: Compositional characterization of PTM scaffolds. Figure S3: Concentration of residual 1,4-dioxane at different lyophilization time and sample temperature. Figure S4: Photos of small scaffolds for the biological evaluation tests. Figure S5: Body weight change of rabbits in the cranial bone regeneration model. Table S1: Grading scale for inflammation scoring. Table S2: Grading scale for histological scoring. Table S3: Criteria for the determination of irritation level of implants. Tables S4–S7: Record sheets of semi-qualitative evaluation for local effects after implantation (week 1–week 26). Table S8: The result of complete blood cell count for subchronic systemic toxicity test. Table S9: The results of clinical chemistry tests for the subchronic systemic toxicity test. Table S10: The results of organ coefficient for the subchronic systemic toxicity test. Tables S11–S16: Record sheets of semi-qualitative evaluation for the rabbit model of cranial bone regeneration (week 2–week 12). Table S17: The specific primers sequences used for real-time PCR.
