# Supplementary material for: Towards the Clinical Translation of 3D PLGA/β-TCP/Mg Composite Scaffold for Cranial Bone Regeneration
Source: Materials (Basel). 2024 Jan 10;17(2):352. doi: 10.3390/ma17020352 (PMC10817297; doi:10.3390/ma17020352)
Supplement: Supplementary file 1 [file materials-17-00352-s001.zip › materials-2793706-supplementary.pdf]

Supporting Information

Towards the clinical translation of 3D PLGA/β-TCP/Mg composite scaffold for cranial bone regeneration

Yongsen Zhou[1,2,3,†], Jingqi Hu[4,†], Binhan Li[1,2,3], Jingjing Xia[1,2,3], Ting Zhang[1,2,3], Zhuo Xiong[1,2,3,*]

[1] Biomanufacturing Center, Department of Mechanical Engineering, Tsinghua University, Beijing, 100084, China

[2] Biomanufacturing and Rapid Forming Technology Key Laboratory of Beijing, Beijing, 100084, China

[3] Biomanufacturing and Engineering Living Systems, Innovation International Talents Base (111 Base), Beijing, 100084, China

[4] National Engineering Research Center of Neuromodulation, School of Aerospace Engineering, Tsinghua University, Beijing 100084, China

[†] These authors contribute equally

* Corresponding author, email: xiongzhuo@tsinghua.edu.cn

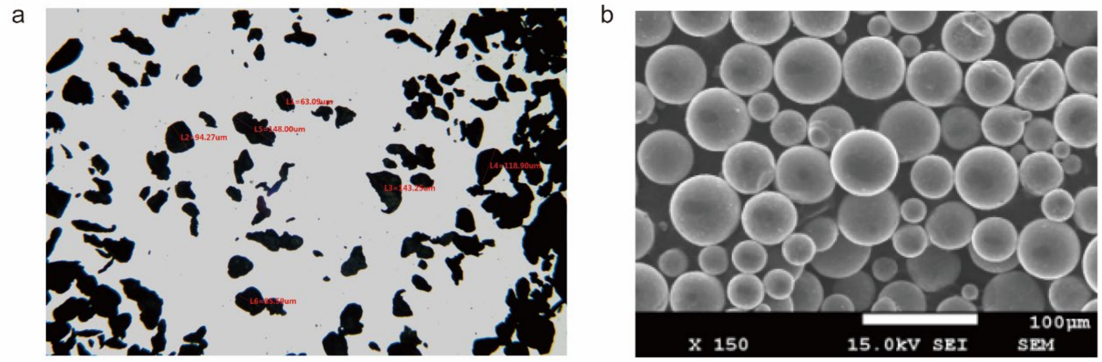

Figure S1. **a** Optical microscope image of Mg powder used in this study. **b** SEM image of Mg powder used in other studies. The SEM image is from the website of supplier (https://www.tswhmf.com/product/9.html).

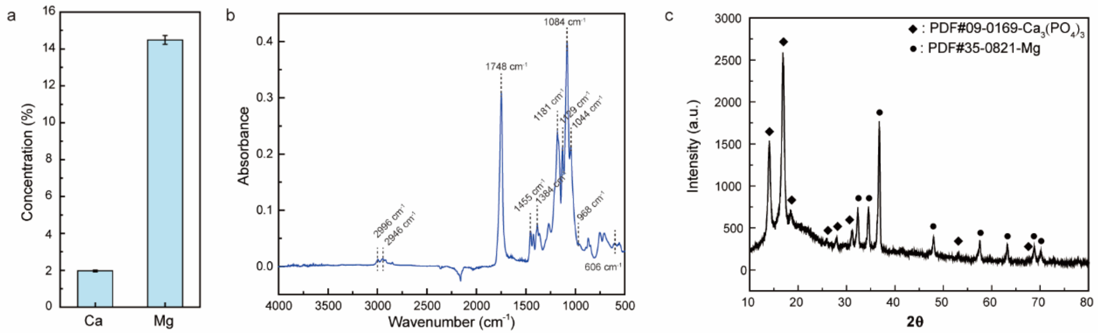

Figure S2. Compositional characterization of PTM scaffolds. **a** Ion concentration detected by ICP-OES. **b** FTIR-ATR spectrum of PTM scaffolds. **c** XRD curves of PTM scaffolds.

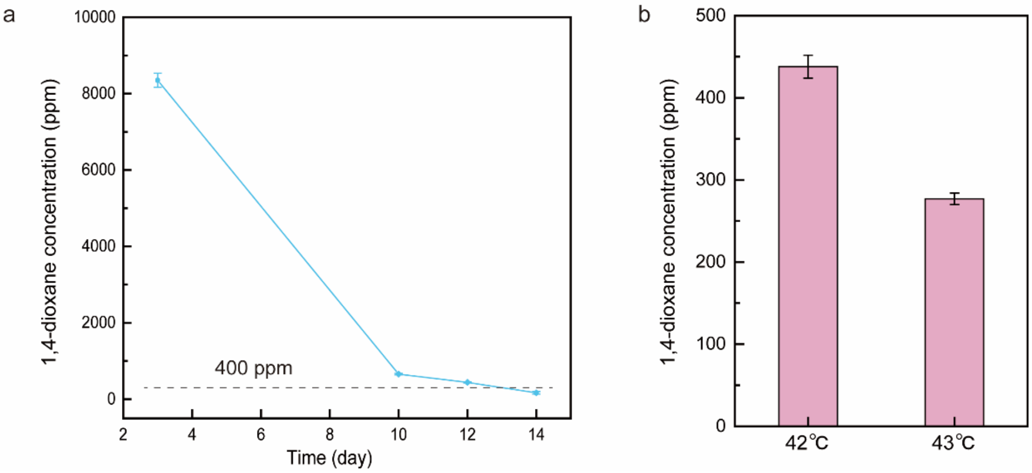

Figure S3. Concentration of residual 1,4-dioxane at different time (a) and temperature (b).

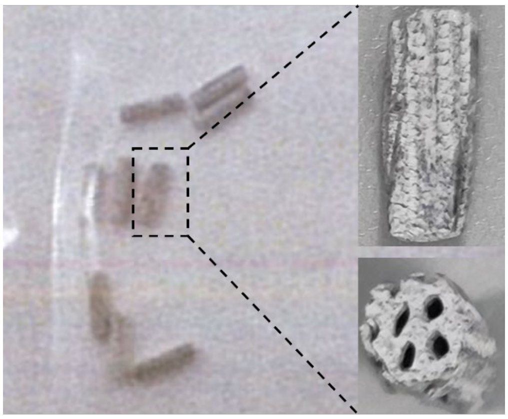

Figure S4. Photos of small scaffolds for the biological evaluation tests.

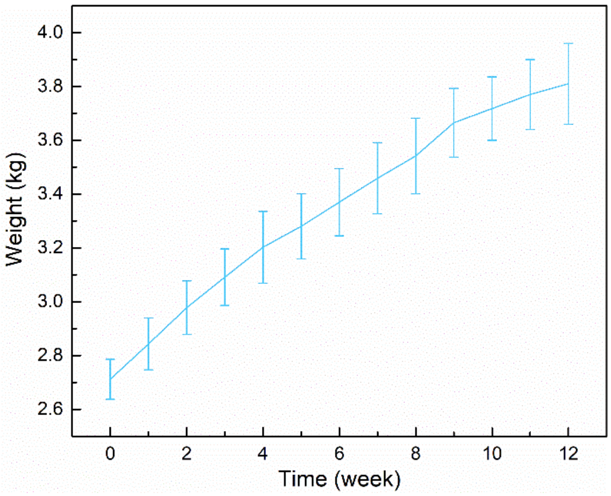

Figure S5. Body weight change of rabbits in the cranial bone regeneration model.

Table S1. Grading scale for inflammation scoring (derived from the ISO 10993-6 Annex E).

**Cell type/response**	**Score**				
	0	1	2	3	4
Neutrophils	0	Rare, 1-5/phf[a]	5-10/phf	Heavy infiltrate	Packed
Lymphocytes	0	Rare, 1-5/phf	5-10/phf	Heavy infiltrate	Packed
Plasma cells	0	Rare, 1-5/phf	5-10/phf	Heavy infiltrate	Packed
Single macrophages	0	Rare, 1-5/phf	5-10/phf	Heavy infiltrate	Packed

Multinucleated giant cells	0	Rare, 1-2/phf	3-5/phf	Heavy infiltrate	Packed
Necrosis	0	Minimal (<25%)	Mild (25-50%)	Moderate (50-75%)	Severe (75-100%)

[a]phf-per high-power field (400 x)

Table S2. Grading scale for histological scoring.

Response	Score				
	0	1	2	3	4
Neovascularization	0	Minor capillaries hyperplasia, focal, 1-3 buds	4-7 groups of capillaries hyperplasia, supplemented by fibroblast structures	Wide range of capillaries hyperplasia, supplemented by fibroblast structures	Extensive capillary hyperplasia, supplemented by fibroblast structures
Fibrosis	0	Restricted areas	Medium thickness area	Thick area	Extensive area
Fat infiltration	0	Minor number of adipose cells, with fibrosis	Several layers of adipose cells, with fibrosis	Adipose cell aggregation area at implant site extended	Implant enclosed by adipose cells

Table S3. Criteria for the determination of irritation level of implants.

Average score of histological examination	0.0-2.9	3.0-8.9	9.0-15.0	>15
Irritation level	None	Mild	Medium	Severe

Table S4. Record sheet of semi-qualitative evaluation for local effects after implantation (week 1).

Sample		PTM scaffolds				Control scaffolds			
Animal number		1	2	3	4	1	2	3	4
Inflammation	Neutrophils	1.00	1.00	1.33	2.00	1.67	1.00	1.67	1.00
	Lymphocytes	0.00	0.00	0.00	0.33	0.33	0.33	0.33	0.33

	Plasma cells	0.00	0.00	0.00	0.00	0.00	0.00	0.00	0.00
	Macrophages	1.00	0.67	0.33	1.33	0.33	1.00	0.33	0.67
	Multinucleated giant cells	0.00	0.33	0.00	0.67	0.00	0.00	0.00	0.00
	Necrosis	0.00	0.00	0.00	0.00	0.00	0.00	0.00	0.00
Cell response total score (×2)		4.00	4.00	3.32	8.66	4.66	4.66	4.66	4.00
Neovascularization		1.00	1.00	1.00	1.00	1.00	1.00	1.33	1.00
Fibrosis		2.33	2.00	2.67	2.33	2.33	3.00	2.33	2.33
Fat infiltration		0.67	0.00	0.33	1.00	0.33	0.67	0.33	0.67
Sum		4.00	3.00	4.00	4.33	3.66	4.67	3.99	4.00
Total score		8.00	7.00	7.32	12.99	8.32	9.33	8.65	8.00
Total score in group		35.31				34.30			
Average		(35.31-34.30)/4=0.25							
Traumatic necrosis		0.00	0.00	0.00	0.00	0.00	0.00	0.00	0.00
Foreign debris		0.00	0.00	0.00	0.00	0.00	0.00	0.00	0.00
Check sites		3	3	3	3	3	3	3	3

Table S5. Record sheet of semi-qualitative evaluation for local effects after implantation (week 4).

Sample		PTM scaffolds				Control scaffolds			
Animal number		5	6	7	8	5	6	7	8
Inflammation	Neutrophils	1.33	1.33	1.00	1.33	1.33	1.00	1.00	1.00
	Lymphocytes	0.67	0.67	0.00	0.33	1.00	0.33	0.67	0.33
	Plasma cells	0.00	0.00	0.00	0.00	0.00	0.00	0.00	0.00
	Macrophages	1.00	1.00	0.67	0.67	0.67	0.00	0.33	0.00
	Multinucleated giant cells	1.00	0.33	0.67	1.00	0.00	0.00	0.00	0.00
	Necrosis	0.00	0.00	0.00	0.00	0.00	0.00	0.00	0.00
Cell response total score (×2)		8.00	6.66	4.68	6.66	6.00	2.66	4.00	2.66
Neovascularization		1.00	1.00	1.00	1.00	1.00	1.00	1.33	1.00
Fibrosis		2.00	2.33	2.00	2.33	2.00	2.00	2.00	2.00
Fat infiltration		0.00	0.00	0.00	0.00	0.00	0.00	0.00	0.00
Sum		3.00	3.33	3.00	3.33	3.00	3.00	3.00	3.00
Total score		11.00	9.99	7.68	9.99	9.00	5.66	7.00	5.66
Total score in group		38.66				27.32			
Average		(38.66-27.32)/4=2.84							
Traumatic necrosis		0.00	0.00	0.00	0.00	0.00	0.00	0.00	0.00
Foreign debris		0.00	0.00	0.00	0.00	0.00	0.00	0.00	0.00
Check sites		3	3	3	3	3	3	3	3

Table S6. Record sheet of semi-qualitative evaluation for local effects after implantation (week 13).

Sample		PTM scaffolds				Control scaffolds			
Animal number		9	10	11	12	9	10	11	12
Inflammation	Neutrophils	1.67	1.33	2.00	1.00	0.67	1.00	0.67	0.67
	Lymphocytes	1.00	1.00	1.00	1.00	0.67	1.00	1.00	0.67
	Plasma cells	0.00	0.00	0.00	0.00	0.00	0.00	0.00	0.00
	Macrophages	0.67	0.67	1.00	1.00	0.00	0.00	0.00	0.33
	Multinucleated giant cells	1.00	0.33	1.00	0.00	0.00	0.00	0.00	0.00
	Necrosis	0.00	0.00	0.00	0.00	0.00	0.00	0.00	0.00
Cell response total score (×2)		8.68	6.66	10.00	6.00	2.68	4.00	3.34	3.34
Neovascularization		1.00	1.00	1.00	1.00	1.00	1.00	1.00	1.00
Fibrosis		2.00	2.00	2.00	3.00	1.67	2.00	1.67	2.00
Fat infiltration		0.00	0.00	0.00	0.00	0.00	0.00	0.00	0.00
Sum		3.00	3.00	3.00	4.00	2.67	3.00	2.67	3.00
Total score		11.68	9.66	13.00	10.00	5.35	7.00	6.01	6.34
Total score in group		44.34				24.70			
Average		(44.34-24.70)/4=4.91							
Traumatic necrosis		0.00	0.00	0.00	0.00	0.00	0.00	0.00	0.00
Foreign debris		0.00	0.00	0.00	0.00	0.00	0.00	0.00	0.00
Check sites		3	3	3	1	3	3	3	3

Table S7. Record sheet of semi-qualitative evaluation for local effects after implantation (week 26).

Sample		PTM scaffolds				Control scaffolds			
Animal number		13	14	15	16	13	14	15	16
Inflammation	Neutrophils	0.33	0.33	0.00	0.33	0.67	1.00	0.67	1.00
	Lymphocytes	0.33	0.67	0.00	0.33	1.00	1.33	0.33	1.00
	Plasma cells	0.00	0.00	0.00	0.00	0.00	0.00	0.00	0.00
	Macrophages	0.00	0.00	0.00	0.00	0.33	0.33	0.00	0.33
	Multinucleated giant cells	0.00	0.00	0.00	0.00	0.00	0.00	0.00	0.00
	Necrosis	0.00	0.00	0.00	0.00	0.00	0.00	0.00	0.00
Cell response total score (×2)		1.32	2.00	0.00	1.32	4.00	5.32	2.00	4.00
Neovascularization		0.33	0.33	0.00	0.67	1.00	1.00	0.67	1.00
Fibrosis		0.67	0.33	0.00	0.33	2.00	2.00	1.67	1.00
Fat infiltration		0.00	0.00	0.00	0.67	0.00	0.00	0.00	0.00
Sum		1.00	0.66	0.00	1.67	3.00	3.00	2.34	2.00

Total score	2.32	2.66	0.00	2.99	7.00	8.32	4.34	6.00
Total score in group	7.97				25.66			
Average	(7.97-25.66)/4=-4.42							
Traumatic necrosis	0.00	0.00	0.00	0.00	0.00	0.00	0.00	0.00
Foreign debris	0.00	0.00	0.00	0.00	0.00	0.00	0.00	0.00
Check sites	3	3	3	3	3	3	3	1

Table S8. The result of complete blood cell count for subchronic systemic toxicity test.

Item	Female SD rats		Male SD rats	
	Experimental group (n=10)	Control group (n=10)	Experimental group (n=10)	Control group (n=10)
WBC (10^9/L)	10.8±2.6**	7.3±2.1	14.4±2.8*	11.3±2.1
RBC (10^{12}/L)	8.0±0.2	7.9±0.3	9.0±0.3	8.8±0.5
HGB (g/L)	150.9±4.2	150.1±3.0	156.7±6.4	155.1±7.2
HCT (%)	43.1±0.8	42.8±0.8	46.1±1.6	45.7±2.5
MCV (fL)	53.9±1.8	54.1±1.5	51.3±1.9	51.7±1.6
MCH (pg)	18.9±0.8	19.0±0.5	17.4±0.7	17.6±0.7
MCHC (g/L)	350.1±5.5	351.0±3.5	340.0±4.7	339.3±5.4
PLT (10^9/L)	1107.8±99.6	1084.8±118.1	1189.1±104.9	1151.3±107.0
NEUT (%)	7.5±2.7*	11.2±3.8	6.5±2.0	8.1±2.2

LYMPH (%)	84.4±4.0*	80.3±3.3	85.8±3.0	82.5±4.3
MONO (%)	7.0±1.7	7.2±0.9	6.7±1.5	8.2±2.3
EO (%)	1.0±0.2	1.2±0.3	0.9±0.4	1.2±0.5
BASO (%)	0.1±0.0	0.1±0.1	0.1±0.0	0.1±0.0
PT (s)	16.2±0.8	16.1±0.5	17.2±0.7	16.7±0.8
APTT (s)	17.0±1.0	16.5±1.6	16.7±1.3	17.8±1.1

WBC: white blood cell. RBC: red blood cell. HGB: hemoglobin. HCT: hematocrit. MCV: mean corpuscular volume. MCH: mean corpuscular hemoglobin. MCHC: mean corpuscular hemoglobin concentration. PLT: platelet count. NEUT: neutrophils. LYMPH: lymphocytes. MONO: monocytes. EO: eosinophils. BASO: basophils. PT: prothrombin time. APTT: activated partial thromboplastin time.

Table S9. The results of clinical chemistry tests for the subchronic systemic toxicity test.

Item	Female SD rats		Male SD rats	
	Experimental group (n=10)	Control group (n=10)	Experimental group (n=10)	Control group (n=10)
ALT (U/L)	34.5±8.3	37.0±11.8	44.9±16.4	39.9±11.8
AST (U/L)	83.4±12.3	91.6±10.6	106.1±32.5	93.8±15.2
TP (g/L)	64.6±5.3	64.3±4.3	58.2±2.1	56.8±3.2
ALB (g/L)	46.1±3.2	44.7±4.5	36.5±1.6	35.6±2.2
TBIL (μmol/L)	2.0±0.5	2.1±0.7	1.5±0.4	1.3±0.3

ALP (U/L)	46.0±13.8	48.4±16.5	105.6±20.6	95.3±18.6
GGT (mmol/L)	-2.2±0.6	-1.9±0.9	-1.9±0.9	-1.5±1.0
GLU (mmol/L)	9.1±1.5**	11.1±1.3	13.1±1.5	11.8±2.7
BUN (mmol/L)	8.0±1.1	7.2±1.4	7.4±1.5	7.0±1.2
CREA (μmol/L)	45.3±6.5	48.0±4.5	46.4±5.5	48.8±5.7
Ca (mmol/L)	2.6±0.1	2.5±0.1	2.5±0.1	2.5±0.2
P (mmol/L)	1.9±0.2	1.8±0.2	2.4±0.2	2.5±0.2
CHOL (mmol/L)	2.0±0.5	1.8±0.5	1.6±0.4	1.6±0.3
TG (mmol/L)	0.4±0.1	0.5±0.1	0.8±0.4	0.5±0.3
K (mmol/L)	4.1±0.4	4.4±0.3	5.0±0.3	5.1±0.4
Na (mmol/L)	138.9±1.1	138.1±1.2	138.7±1.1	139.0±1.8
Cl (mmol/L)	102.0±2.0	101.2±1.3	99.7±1.7	99.9±2.0

ALT: alanine transaminase. AST: aspartate transferase. TP: total protein. ALB: albumin. TBIL: total bilirubin. ALP: alkaline phosphatase. GGT: gamma-glutamyl transferase. GLU: glucose. BUN: blood urea nitrogen. CREA: creatinine. CHOL: cholesterol. TG: triglyceride.

Table S10. The results of organ coefficient (organ weight/100g body weight) for the subchronic systemic toxicity test.

Item	Female SD rats		Male SD rats	
	Experimental group (n=10)	Control group (n=10)	Experimental group (n=10)	Control group (n=10)
Heart	0.3421±0.0308	0.3317±0.0288	0.2626±0.0179	0.2626±0.0279
Liver	2.5360±0.1792	2.4847±0.2385	2.4879±0.2561	2.5002±0.2947
Spleen	0.1770±0.200	0.1620±0.0316	0.1382±0.0132	0.1345±0.0183
Lung	0.4712±0.0577	0.4734±0.0664	0.3630±0.0695	0.3458±0.0387
Kidney	0.6186±0.0341	0.6422±0.0645	0.5478±0.0324	0.5713±0.0574
Adrenal	0.0229±0.0049	0.0262±0.0045	0.0111±0.0029	0.0115±0.0028
Ovary & uterus	0.2880±0.0560	0.3007±0.0404	/	/
Testicle & epididymis	/	/	0.8815±0.2293	1.0152±0.1072
Thymus	0.0941±0.0155	0.1065±0.0283	0.0872±0.0219	0.0731±0.0208
Brain	0.6468±0.0751	0.6474±0.0454	0.3626±0.0266	0.3725±0.0315
Stomach	0.5660±0.0871	0.5353±0.0337	0.4049±0.0613	0.4094±0.0311

Table S11. Record sheet of semi-qualitative evaluation for the rabbit model of cranial bone regeneration (week 2).

Sample		PTM scaffolds		Blank	
Animal number		7200	7201	7200	7201
Inflammation	Neutrophils	0.00	0.00	0.00	0.00

	Lymphocytes	1.00	1.00	0.50	1.00
	Plasma cells	0.00	0.00	0.00	0.00
	Macrophages	1.50	2.00	1.00	1.00
	Multinucleated giant cells	1.00	0.00	0.00	0.00
	Necrosis	0.00	0.00	0.00	0.00
Cell response total score (×2)		7.00	6.00	3.00	4.00
Neovascularization		1.00	1.00	1.00	1.00
Fibrosis		1.00	1.00	1.00	1.00
Fat infiltration		0.00	0.00	0.00	0.00
Sum		2.00	2.00	2.00	2.00
Total score		9.00	8.00	5.00	6.00
Total score in group		17.00		11.00	
Average		(17.00-11.00)/2=3.00			
Traumatic necrosis		0.00	0.00	0.00	0.00
Foreign debris		0.00	0.00	0.00	0.00
Check sites		2	2	2	2

Table S12. Record sheet of semi-qualitative evaluation for the rabbit model of cranial bone regeneration (week 4).

Sample		PTM scaffolds		Blank	
Animal number		7202	7203	7202	7203
Inflammation	Neutrophils	0.00	0.00	0.00	0.00
	Lymphocytes	1.00	1.00	0.50	0.00
	Plasma cells	0.00	0.00	0.00	0.00
	Macrophages	1.50	1.00	1.00	1.00
	Multinucleated giant cells	0.00	0.00	0.00	0.00
	Necrosis	0.00	0.00	0.00	0.00
Cell response total score (×2)		5.00	4.00	3.00	2.00
Neovascularization		1.00	1.00	1.00	1.00
Fibrosis		1.00	1.00	1.00	1.00
Fat infiltration		0.00	0.00	0.00	0.00
Sum		2.00	2.00	2.00	2.00
Total score		7.00	6.00	5.00	4.00
Total score in group		13.00		9.00	
Average		(13.00-9.00)/2=2.00			
Traumatic necrosis		0.00	0.00	0.00	0.00
Foreign debris		0.00	0.00	0.00	0.00
Check sites		2	2	2	2

Table S13. Record sheet of semi-qualitative evaluation for the rabbit model of cranial bone regeneration (week 6).

Sample		PTM scaffolds		Blank	
Animal number		7204	7205	7204	7205
Inflammation	Neutrophils	0.00	0.00	0.00	0.00
	Lymphocytes	1.00	0.00	0.00	0.00
	Plasma cells	0.00	0.00	0.00	0.00
	Macrophages	0.50	1.00	0.00	0.00
	Multinucleated giant cells	0.50	0.00	0.00	0.00
	Necrosis	0.00	0.00	0.00	0.00
Cell response total score (×2)		4.00	2.00	0.00	0.00
Neovascularization		2.50	2.00	0.50	2.00
Fibrosis		2.50	2.50	1.00	2.50
Fat infiltration		0.00	0.00	0.00	0.00
Sum		5.00	4.50	1.50	4.50
Total score		9.00	6.50	1.50	4.50
Total score in group		15.50		6.00	
Average		(15.50-6.00)/2=4.75			
Traumatic necrosis		0.00	0.00	0.00	0.00
Foreign debris		0.00	0.00	0.00	0.00
Check sites		2	2	2	2

Table S14. Record sheet of semi-qualitative evaluation for the rabbit model of cranial bone regeneration (week 8).

Sample		PTM scaffolds		Blank	
Animal number		7206	7207	7206	7207
Inflammation	Neutrophils	0.00	0.00	0.00	0.00
	Lymphocytes	2.00	1.00	0.00	0.00
	Plasma cells	0.00	0.00	0.00	0.00
	Macrophages	0.00	0.00	0.00	0.00
	Multinucleated giant cells	0.00	0.00	0.00	0.00
	Necrosis	0.00	0.00	0.00	1.50
Cell response total score (×2)		4.00	2.00	0.00	3.00
Neovascularization		1.50	2.00	1.50	3.00
Fibrosis		2.50	2.00	0.00	0.00
Fat infiltration		0.00	0.00	0.00	0.00
Sum		4.00	4.00	1.50	3.00
Total score		8.00	6.00	1.50	6.00
Total score in group		14.00		7.50	
Average		(14.00-7.50)/2=3.25			
Traumatic necrosis		0.00	0.00	0.00	0.00

Foreign debris		0.00	0.00	0.00	0.00
Check sites		2	2	2	2

Table S15. Record sheet of semi-qualitative evaluation for the rabbit model of cranial bone regeneration (week 10).

Sample		PTM scaffolds		Blank	
Animal number		7208	7209	7208	7209
Inflammation	Neutrophils	0.00	0.00	0.00	0.00
	Lymphocytes	0.00	0.00	0.50	0.50
	Plasma cells	0.00	0.00	0.00	0.00
	Macrophages	0.00	0.00	0.00	0.50
	Multinucleated giant cells	0.00	0.00	0.00	0.00
	Necrosis	0.00	0.00	0.00	0.00
Cell response total score (×2)		0.00	0.00	1.00	2.00
Neovascularization		0.50	0.50	0.50	0.50
Fibrosis		1.00	1.00	2.00	1.50
Fat infiltration		0.00	0.00	0.00	0.00
Sum		1.50	1.50	2.50	2.00
Total score		1.50	1.50	3.50	4.00
Total score in group		3.00		7.50	
Average		(3.00-7.50)/2=-2.25			
Traumatic necrosis		0.00	0.00	0.00	0.00
Foreign debris		0.00	0.00	0.00	0.00
Check sites		2	2	2	2

Table S16. Record sheet of semi-qualitative evaluation for the rabbit model of cranial bone regeneration (week 12).

Sample		PTM scaffolds		Blank	
Animal number		7210	7211	7210	7211
Inflammation	Neutrophils	0.00	0.00	0.00	0.00
	Lymphocytes	0.50	0.00	1.50	0.50
	Plasma cells	0.00	0.00	0.00	0.00
	Macrophages	0.00	0.00	0.00	0.00
	Multinucleated giant cells	0.00	0.00	0.50	0.00
	Necrosis	0.00	0.00	0.00	0.00
Cell response total score (×2)		1.00	0.00	4.00	1.00
Neovascularization		0.50	0.00	0.50	0.00
Fibrosis		1.50	0.00	1.50	0.00
Fat infiltration		0.00	0.00	0.00	0.00

Sum	2.00	0.00	0.00	0.00
Total score	2.00	0.00	6.00	1.00
Total score in group	3.00		7.00	
Average	(3.00-7.00)/2=-2.00			
Traumatic necrosis	0.00	0.00	0.00	0.00
Foreign debris	0.00	0.00	0.00	0.00
Check sites	2	2	2	2

Table S17. The specific primers sequences used for real-time PCR.

Primer	forward	reverse
GADPH	3’-CATGTTCCAGTATGACTCCACTC-5’	3’-GGCCTCACCCCATTTGATGT-5’
BMP2	3’-GCTCCACAAACGAGAAAAGC-5’	3’-AGCAAGGGGAAAAGGACACT-5’
RUNX-2	3’-AGACTGCAAGAAGGCTCTGG-5’	3’-TTCCTGCATGGACTGTGGTT-5’
OCN	3’-CTGACCTCACAGATCCCAAGC-5’	3’-TGGTCTGATAGCTCGTCACAAG-5’
OPN	3’-CCAACGGCCGAGGTGATA-5’	3’-CAGGCTGGCTTTGGAACTTG-5’
BSP	3’-CAGGGAGGCAGTGACTCTTC-5’	3’-AGTGTGGAAAGTGTGGCGTT-5’
OPG	3’-AAAGCACCCTGTAGAAAACA-5’	3’-CCGTTTTATCCTCTCTACACTC-5’
